# Treatment with a vascular disrupting agent does not increase recruitment of indium labelled human endothelial outgrowth cells in an experimental tumour model

**DOI:** 10.1186/1471-2407-14-903

**Published:** 2014-12-02

**Authors:** Lotte B Bertelsen, Anja B Bohn, Yuan Yuan Shen, Lise Falborg, Hans Stødkilde-Jørgensen, Michael R Horsman

**Affiliations:** MR-Research Centre, Department of Clinical Medicine, Aarhus University Hospital, Aarhus, Denmark; Department Experimental Clinical Oncology, Aarhus University Hospital, Aarhus, Denmark; The Affiliated Hospital of Binzhou Medical University, Binzhou, Shandong China; Department of Nuclear Medicine and PET Centre, Aarhus University Hospital, Aarhus, Denmark

**Keywords:** Endothelial outgrowth cells, Vascular disrupting agents, CA4P, Tumour

## Abstract

**Background:**

The effect of vascular disrupting agents in tumour therapy depends on both the immediate vascular shutdown, and on the following re-vascularization of the tumour. The aim of this study was to use a tumour model to investigate whether endothelial outgrowth cells (EOCs) influenced the short term treatment efficiency of combretastatin A-4 disodium phosphate (CA4P) and 5,6-dimethylxanthenone-4-acetic acid (DMXAA) by increasing EOC tumour recruitment.

**Methods:**

In order to visualize the recruitment of EOCs to the tumours, umbilical cord blood derived human EOCs were labelled with ^111^Indium-tropolone in a dose of 0.37 MBq pr 3×10^6^ cells and were injected intravenously into mice carrying a C3H mammary carcinoma on their right rear foot. DMXAA and CA4P in different concentrations and at different exposure times were used to create a hypoxic environment in the C3H mammary carcinoma in the mice. Three different mice strains with various degrees of functional immune system were used to study the homing capability of EOCs.

**Results:**

Our data showed that approximately 4% of the total injected radioactive dose per gram of tissue was found in the tumour after treatment with CA4P and DMXAA. Regardless of the concentration and the treatment duration, CA4P did not increase EOC recruitment to the tumour in comparison to EOC recruitment in control tumours in any of the 3 mice strains studied.

**Conclusion:**

Our data showed that regardless of the grade of the immune system, ranging from a fully working to a fully compromised immune system, treatment with CA4P did not increase recruitment of xenotransplanted EOCs to tumour tissue.

## Background

Endothelial Progenitor Cells (EPCs) are a source of cells that can participate in vasculogenesis [1]. They are able to circulate, proliferate, and participate in the development of a vascular network [2, 3] as well as augmenting angiogenesis through the secretion of angiogenic growth factors [4, 5]. Endothelial progenitor cells are a heterogeneous population which can be divided into several subpopulations, which may differ in origin and function [5, 6].The EPCs are mobilised in response to tissue ischemia [7] and may be involved in re-vascularization of tumour tissue following incomplete tumour treatment [3, 8, 9]. The endothelial outgrowth cells (EOC) are a subtype of EPCs that have clonogenic and proliferative potential [10–12] and has been shown to increase tumour growth rates [13].

For data on bio-distribution the radioactive agent ^111^Indium (^111^In) is frequently used and in vivo studies has earlier been performed with indium labelled EPCs [14, 15] as this labelling technique is able of showing the dynamic distribution properties in organs.

Tumours are characterised by a vascular network that consists of immature blood vessels with large fenestrations in the endothelial cell layer and in the basal membrane [16]. When tumours reach a size of approximately – 1 mm^3^ further expansion depends critically on the development of new vessels to maintain oxygen and nutrient delivery [17, 18]. The demand of new vessel formation for expansion of tumour tissue has made the vasculature an obvious target for anti-tumour therapy both in the form of anti-angiogenic pharmaceuticals and by vascular disrupting agents (VDA) [19]. These drugs inhibit formation of new blood vessels and target the existing vasculature in tumours, respectively [19, 20]. VDAs are known primarily to mediate their effects on tumour blood vessels resulting in a transient effect of therapy with tumour hypoxia, ischemia, and cell death as a result [19–23]. In this study we use the VDA compounds Combretastatin A-4 disodium phosphate (CA4P) and 5,6-dimethylxanthenone-4-acetic acid (DMXAA) both of which selectively mediate vascular shutdown of existing vasculature inside solid tumors [24, 25]. However these drugs exert their anti-vascular action in different approaches. CA4P is a tubulin binding agent that inhibit microtubule assembly [26] and it has been shown to induce a peak in peripheral blood Endothelial Progenitor Cells (EPC) concentration within 4 hours after treatment [8, 9]. DMXAA belongs to the group of flavonoids which induce secretion of cytokines [27] and recruitment of immune cells [28].

The hypoxia occurring after VDA treatment may stimulate the release of EPCs from the bone marrow and thereby the recruitment of EPCs to the tumour in order to increase the blood supply to the necrotic areas [29–32]. However, it is not fully settled to which extent EPC recruitment to the tumour occurs, therefore the aim of the present study was to analyse the degree of homing of xenotransplanted ^111^Indium (^111^In) labelled EOCs in tumour bearing mice after treatment with the VDA compounds CA4P and DMXAA.

## Methods

### Animal and tumour models

All animal procedures were approved and conducted in accordance with the institutional guidelines for the care of laboratory animals and with the Danish Animal Experiments Inspectorate’s approval. This study was performed on female CDF1, NMRI-nu/nu mice, and CIAE-NOG mice obtained from Taconic Laboratories (Ry, Denmark). Ten-fourteen week-old mice were implanted with a C3H mammary carcinoma subcutaneously in the right rear foot. The derivation and maintenance of this tumour have been described in detail previously [33]. Experiments were performed when tumours reached approximately 200 mm^3^ in size, which typically occurred 3 weeks after inoculation. All experiments were performed using non-anesthetized animals.

### Drugs

The pro-drug CA4P and the active drug combretastatin A4 (CA4) were supplied by OXiGENE, Inc. (Waltham, MA, USA). DMXAA was provided by Dr. William Denny at the University of Auckland, New Zealand. The drugs were prepared fresh before each experiment, by dissolving in saline, and were kept cold and protected from light until used. For in vivo experiments, CA4P was injected intraperitoneally (i.p.) in one of the following doses; 25 mg/kg or 250 mg/kg. For cell viability studies, CA4P and CA4 were used in a concentration range of 0.1 to 10 μM. DMXAA was injected i.p. at a dose of 20 mg/kg.

### EOC cultures

Mononuclear cells (MNCs) were isolated from human umbilical cord blood and the umbilical cord blood samples were collected from fresh placentas. The collection and experimental procedure was performed with written consent from the mothers and with permission from the local ethical committee (Videnskabsetisk komité Region Midtjylland, 1988/1349). MNCs were isolated by density gradient centrifugation using ficoll paque plus (400 × g for 30 minutes (min)) after a 1:3 dilution with phosphate buffered saline (PBS). After three washes in PBS, CD34 positive cells were purified using magnetic cell separation (MACS) with CD34+ microbeads and LS columns (Miltenyi Biotec, Lund, Sweden). Cells were seeded at a density of 1–1.8 × 10^6^ cells/2 cm^2^ on gelatin (Sigma-Aldrich, Brondby, Denmark) coated 12 well plates and were maintained in endothelial basal medium-2 (EBM-2) supplemented with EGM-2 mv Single-Quots (EGM-2 mv medium, Lonza, Walkersville, MD, USA) for four days. After day 4, the medium was changed every day for the following week. For the rest of the culture period the medium was changed every second day. Colonies with cobblestone-like morphology appeared after 2–3 weeks in culture. These cells were cultured until they formed larger colonies. These colonies were expanded over several passages by use of standard cell culture procedures. All cells were maintained under standard conditions (5% CO_2_, 37°C).

Endothelial progenitor cells that had gone through 4–6 passages were analysed for a specified panel of cell surface markers using Flow Cytometry (FACS Calibur or FACSCanto™II, BD Biosciences, Brondby, Denmark) and Flow Jo (version 9.3.1, Tree Star Inc., Ashland, OR). Cells with a cell surface antigen profile of CD34+, CD45- and, CD133- were considered to be endothelial outgrowth cells as shown in other studies [34].

### Cell proliferation and viability studies

For determination of cell proliferation and cell viability upon treatment with CA4 and CA4P, EOCs were grown at 3.4 × 10^4^ cells/2 ml EGM-2mv medium. The cells were treated with either medium (control) or 0.1, 0.3, 1, 3 or 10 μM of either CA4 or CA4P and were incubated at 5% CO_2_, 37°C for two hours. After incubation the medium was changed. Cells were supplemented with fresh medium after 2 and 4 days and were harvested either 2, 4, or 6 days after VDA treatment. The cell count and viability were determined using the Trypan blue exclusion test.

### Radioactive labelling

EOCs were detached from culture flasks using trypsin and were centrifuged (480 × g, 5 min). The cell suspension was incubated with ^111^ In-tropolone for 15 min at 37°C at a labelling dose of 0.37 MBq/3 × 10^6^ cells. After the incubation period, the cells were washed twice in PBS and centrifuged at 400 × g for 10 min, before administration into the mice. The details of EOC labelling, retention and incorporation into the vasculature have been described before [35].

### Cell transplantation

Before transplantation, the ^111^In labelled cells were suspended in a concentration of 3 × 10^6^ cells/200 μl PBS immediately before injection into recipient tumour-bearing mice. Immune-competent, CDF1 mice (n = 5 in each of 9 groups) and immune-deficient, NMRI-nu/nu mice (n = 5 in each of 4 groups) and CIAE-NOG mice (n = 5 in each of 3 group) were placed in a jig and cells were injected into the tail vein of the mouse. Each mouse received 200 μl cell suspension by intravenous injection.

### Radioactivity tracing in mice

Twenty-four hours after injection of the ^111^In-tropolone labelled cells, a sample of 150 μl blood was taken into dry EDTA tubes from the sub-orbital sinus of each mouse. Immediately after blood samples were taken, the mice were killed by cervical dislocation and samples of the tumour, heart, lung, liver, kidney, spleen, muscle, bone, brain, intestine, and bladder were excised and weighed before measuring the radioactivity using a gamma counter (Packard, Canberra Company, GMI, Minnesota, USA). The radioactivity of the carcass of the mice was measured using a dosi-calibrator (Amersham, Capintec Inc., New jersey, USA). The specific activity in the organs was calculated as percent of the injected radioactive dose per gram of tissue (%ID/g) after correction for radioactive decay.

### Statistics

Results are presented as mean ± 1 SE. A Student’s t-test or Mann–Whitney Rank Sum Test were used for pair wise comparison and a one way ANOVA was used for multiple comparisons between groups (Sigma plot version 12). P < 0.05 was considered to indicate a significant difference.

## Results

### In vitro toxicity assay

**Figure 1:**
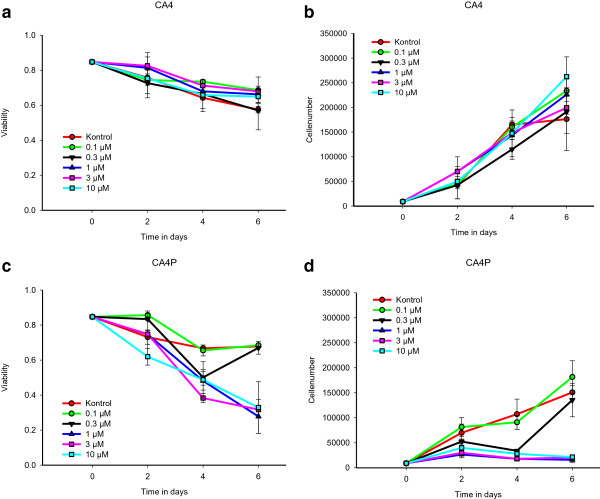
**In vitro effect of 2 hour incubation of EOCs in the presence of CA4 or CA4P.** EOCs were incubated for 2 hours in the presence of 0, 0.1, 0.3, 1, 3, or 10 μM CA4 or CA4P. After two hours fresh medium was added and the cells were cultured for additional 2, 4 or 6 days. **(a)** and **(c)** Viability as a function of time in days in EOC cell cultures initially treated with different concentrations of CA4 or CA4P. **(b)** and **(d)** Cell number as a function of time in days in EOC cell cultures initially treated with different concentrations of CA4 and CA4P. The initial starting value was a total of 9000 cells. Data is presented as mean ± 1 SE (n = 2 in each group).

To investigate the effect of CA4P on EOCs, we treated EOCs in culture with medium (control) or different doses of either the active compound CA4 or the pro-drug CA4P for 2 hours and followed the cell cultures for up to 6 days after treatment with VDA. As shown in Figure 1a and c, we observed a decrease in cell viability over time in both control groups as well as in all the examined CA4 concentration. This decrease, as shown in Figure 1b, corresponded well with a high increase in cell number in the cell culture wells, resulting in limited capacity for the cells to adhere. This confluence provided suboptimal cell conditions and to some extent cell death. Moreover CA4P concentrations higher than 1 μM resulted in decreased cell viability over time as well as a total inhibition in their proliferation potential over time as shown in Figure 1 c and d.

### Distribution of ^111^In labelled EOCs in different mice strains

To gain information about ^111^In labelled EOCs in a tumour model and clearance of these xenotransplanted cells by the immune system, we administered ^111^In labelled human EOCs into the tail vein of 3 different mouse strains and investigated the tracer distribution 24 hours after administration.

**Figure 2:**
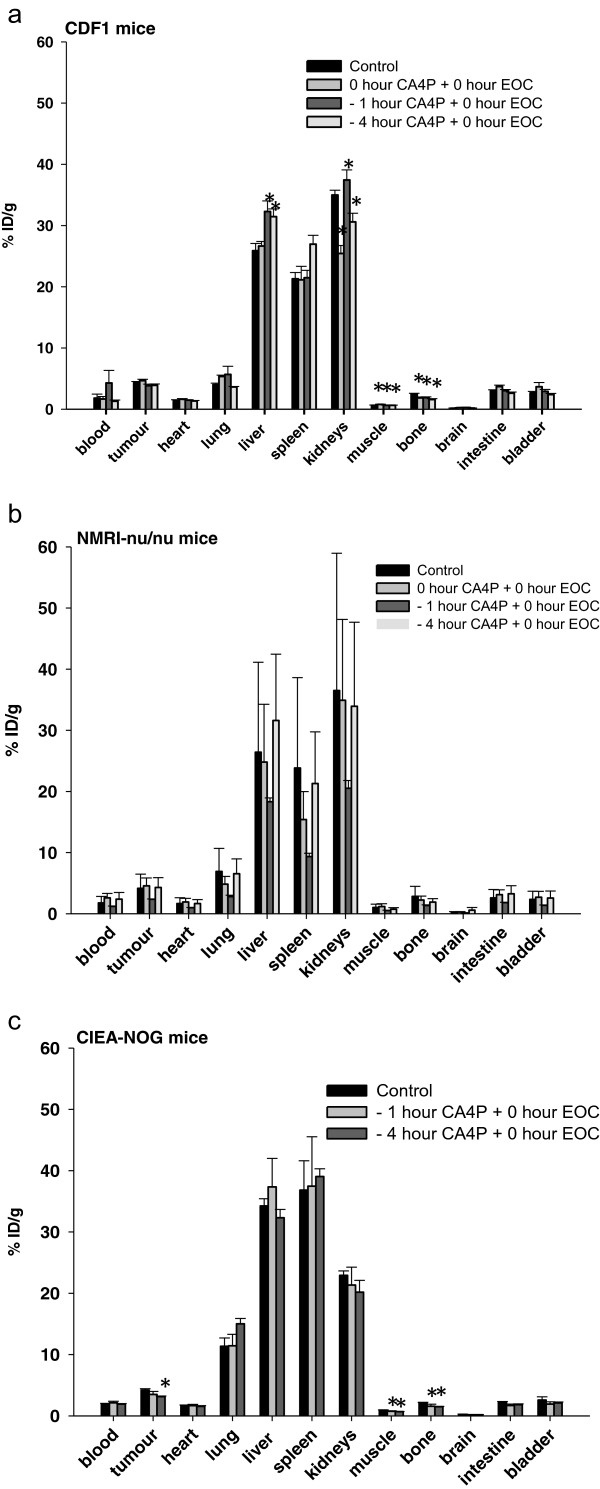
**Distribution of** ^**111**^ **In labelled EOCs after treated of mice with 25 mg/kg CA4P.** Tissue distribution of radioactivity after i.v. injection of ^111^In labelled EOCs in **(a)** CDF1 mice, **(b)** NMRI-nu/nu and **(c)** CIAE-NOG mice. The mice were treated with CA4P at different time points before administration of ^111^In labelled EOCs and control mice received only labelled EOCs. All mice were sacrificed 24 hours after experimental start. Data is presented as mean ± 1 SE (n = 5 in each group). A one way ANOVA were used for comparison between the different mice strains and between the control group and different CA4P treatment groups in each organ in each mouse strain.

Comparing the different mice strains, with immune systems ranging from fully working to a fully compromised immune system, Figure 2 shows, that the distribution pattern of the ^111^In labelled cells is merely the same for control mice in all three mice strains. Most of the ^111^In activity is located in the liver, spleen, and kidneys. Though non-significant, there is a small difference in the amount of radioactivity in the spleen and the kidney- with the fully immune compromised mice (CIEA-NOG) having more ^111^In accumulated in the spleen and less in the kidneys compared to the two other mice strains.

### Distribution of ^111^In labelled EOCs after treatment with 25 mg/kg CA4P

To determine the effect of CA4P treatment on the distribution of ^111^In labelled EOCs in a tumour model, we administered ^111^In labelled human EOCs into the tail vein of 3 different mouse strains and investigated the tracer distribution 24 hours after 25 mg/kg CA4P and EOC administration. Overall, a similar amount of radioactive EOCs was found (Figure 2) in the tumour in the 3 different mouse strains used in this experiment. In the control mice and mice treated with CA4P and EOCs at the same time, the amount of radioactivity in the tumours corresponded to 4% ID/g ≈ 0.5% of the injected EOCs, or ≈ 1.5 ×10^4^ EOCs.

To evaluate the optimal time-point for injecting EOCs we injected these cells at various time points after CA4P treatment in all three mice strains. As seen in Figure 2a, b, and c, in the mice receiving CA4P 1 and 4 hours before EOC administration, the amount of radioactivity in the tumours changed slightly, however ^111^In accumulation in tumours was only significantly different from controls in CIEA-NOG mice receiving the CA4P 4 hours before EOC administration.

### Distribution of ^111^In labelled EOCs after treatment with 250 mg/kg CA4P and 20 mg/kg DMXAA

**Figure 3:**
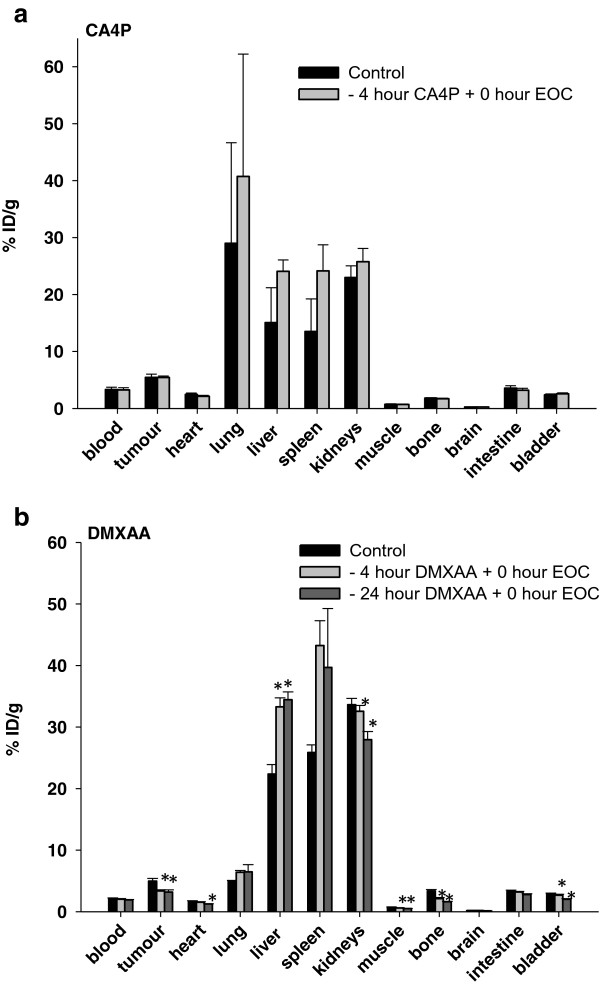
**Distribution of** ^**111**^ **In labelled EOCs after treatment of mice with 250 mg/kg CA4P or 20 mg/kg DMXAA.** Tissue distribution of ^111^In radioactivity in different tissues after i.v. injection of ^111^In labelled EOCs in CDF1 mice. **(a)** Control mice receiving only labelled EOCs or mice treated with 250 mg/kg CA4P 4 hours prior to EOC administration. **(b)** Control mice receiving only labelled EOCs or mice treated with 20 mg/kg DMXAA 4 hours or 24 prior to EOC administration. Data is presented as mean ± 1 SE (n = 5 in each group).

As listed in Table 1, the necrotic fraction in tumours from mice treated with 25 mg/kg CA4P was higher than the necrotic fraction in control mice, but yet small. A higher dose of CA4P (250 mg/kg) increased the necrotic fraction as compared to treatment with both 25 mg/kg CA4P, and to the control tumours. DMXAA (20 mg/kg) mediated the highest amount of tumour necrosis. For that reason, and in order to induce a higher EOC recruitment to tumours, we treated the mice with either a higher dose of CA4P, (250 mg/kg) 4 hours before EOC administration (Figure 3a) or with DMXAA (20 mg/kg) 4 hours before cell administration (Figure 3b). As seen from Figure 3a and b, the distribution of the ^111^In signal from all the examined organs was in the same range as observed in animals treated with the low dose of CA4P (Figure 2a). Figure 3a and b illustrates that neither CA4P, (250 mg/kg), nor DMXAA (20 mg/kg) treatment caused a higher EOC-recruitment to tumours compared to tumours in control mice. In contrast, treatment with DMXAA resulted in a significant lower amount of ^111^In in the tumours when compared to the control mice.

Additionally, we analysed whether an increased timespan for the DMXAA to mediate its effect changed the recruitment of the labelled EOCs to the tumour. As shown in Figure 3b, mice receiving DMXAA 4 hours and 24 hours before EOCs had the same recruitment of EOCs to the tumour site and this was significantly lower than the EOC tumour recruitment in the control mice.

**Table 1:** **Necrotic fraction in C3H tumours in control and VDA treated mice**

Treatment	Necrotic fraction
Control	18.8 ± 1.4*
CA4P (25 mg/kg)	25.4 ± 2.6*
Control	9.3 ± 1.9^#^
CA4P (250 mg/kg)	37.0 ± 4.3^#^
Control	7.4 ± 3.4^§^
DMXAA (20 mg/kg)	77.3 ± 4.5^§^

CA4P: Combrestatin A-4 disodium phosphate; DMXAA: 5,6-dimethylxanthenone-4-acetic acid; *Anja B. Bohn, Lotte B. Bertelsen and Thomas Wittenborn unpublished data; ^#^Murata et al. [22], Radiother. Oncol. 60: 155–161; ^§^Murata et al. [36], Int. J. Hyperthermial. 17: 508–519. Data is presented as mean ± SE.

## Discussion

It has been believed for decades that EOCs have the potential to home to sites of hypoxia in response to signals given from that area [31, 37, 38]. CA4P induce hypoxia [39] and therefore the recruitment of EOCs to the tumour could be increased after CA4P treatment in order for EOCs to help in the angiogenic progression [8, 9, 25]. In the present study, we determined the effect of CA4P treatment on EOC recruitment to the tumour in 3 different mice strains. We confirm a previous study showing the presence of EOCs in the tumour 24 hours after administration of these human derived EOCs [35], but, we did not observe an increased recruitment of EOCs to the tumour after CA4P treatment.

It has previously been shown that CA4 and CA4P is toxic towards proliferating cells [21]. We analysed the effect of CA4, as well as its pro-drug CA4P on viability and proliferation of EOCs in vitro and our results supported these previous findings. Using the active compound CA4, we observed a decreased viability; however, this decrease could be explained by confluency in the cell culture resulting in cell death for some of the cells. Using CA4P, in concentrations higher than 1 μM, decreased cell viability over time and mediated a total arrest in their proliferation potential.

Although the in vitro results indicate potential toxicity of CA4P towards EOCs, this effect may not be observed in vivo as this pro-drug is rapidly cleaved into CA4 by nonspecific endogenous phosphatases, present in plasma [40] and is therefore in its active form before convention with the EOCs.

Mobilization and functional incorporation of EPCs into the tumour vasculature is essential for the growth of some tumours, however, some tumour types or different tumour grade and stage may be more dependent on EPCs for their growth than other [38]. Therefore, we examined if CA4P treatment induced recruitment of xenotransplanted EOCs to the tumour tissue. The C3H mammary carcinoma used in these studies has been shown to be comparable to clinical studies when studying the effect of CA4P [41]. Earlier studies have also shown that VDA treatment causes a peak in circulating EPCs 4 hours after administration [8] and furthermore a peak of EPCs was found in the blood and the tumour of CA4P treated mice [9]. Therefore, we expected the CA4P-mediated vascular damage to result in an increased infiltration of human EOCs in the tumour. However, we found no significant increase in EOC recruitment at any of the VDA doses or treatment intervals used, when compared to control mice. Contradictory when examining, fully immune-competent mice (CDF1 mice), mice with an absent thymus (NMRI-nu/nu mice) and super immune deficient mice (CIEA-NOG mice), we found nearly the same degree of EOC recruitment to the tumour in control and VDA treated mice. However, in the tumours of CIEA-NOG mice we observed a small decrease in EOC recruitment in CA4 treated mice compared to control mice, and when these mice were treated with CA4P 4 hours prior to cell administration this decrease was significant. In immune-competent mice, cells like monocytes/-macrophages and T lymphocytes, participate in the angiogenic process by secreting pro- and anti-inflammatory cytokines, that could control endothelial cell proliferation, their survival and apoptosis, as well as their migration and activation [42]. The decreased recruitment of EOCs in the tumour of CIEA-NOG mice could be caused by the lack of functional T, B and NK cells and deficiencies in signalling, including impaired cytokine production in these mice. This could result in impaired signalling to the murine endothelial cells as well as the xenotransplanted EOCs and therefore decreased recruitment of these cells to the tumour.

As mentioned above, we tested the recruitment of human derived EOCs in 3 different mice strains with different degrees of operational immune systems. During the time span examined, we found nearly the same degree of EOC recruitment to the tumour exposed to a 25 mg/kg CA4P as to tumours in control mice independent of mice strains. A higher dose of CA4P was therefore chosen in the CDF1 mice. Increasing the CA4P dose to 250 mg/kg did, however, did not change the EOC recruitment to tumours. These data indicated that either the CA4P mediated vascular injury was not causing a compensating angiogenesis post treatment or that EOCs are not able to reach the vessels to mediate the development of new vessels within the time span examined. Hence, in order to induce more vascular damage, we used the VDA compound DMXAA, which induces a higher fraction of necrosis in the C3H tumour (Table 1), and thus, theoretically could cause recruitment of a larger pool of EOCs to the tumour. Despite the fact that EOCs are believed to home to necrotic tumour areas [2, 13, 43], we did not observe an increased recruitment of these cells to the tumour in response to any of the VDA treatments used in this study. On the contrary, we observed a decreased recruitment of EOCs when the necrotic fraction in the tumours increased. We cannot exclude the possibility that either the EOCs are somehow being prevented from reaching the tumour areas, or that the tumour vessels preferentially are recruiting murine EPCs and that these host cells are sufficient to fulfil the need of vessel formation.

Taylor et al. showed an increased amount of murine EPCs in a tumour model, different from ours, days after CA4P treatment. However, as several studies has shown variable extent of EPC participation in tumour vasculature [38] the use of different tumour type, grade, and sizes makes it difficult to compare EPC recruitment across studies. In this and a previous study we have shown that xenotransplanted EOCs can be found in the tumour 24 hours after injection. The cells were, however, not found in the endothelial lining of small vessels in the tumour, but were present in the tumour rim where they might have a paracrine signalling function.

In this study, we also show that most of the ^111^In activity was found in the liver, spleen, and kidneys in all 3 mice strains. The most immune-compromised animal (CIEA-NOG), had accumulated indium activity in the spleen and less in the liver compared to the fully immune competent mice (CDF1 mice) and mice with an absent thymus (NMRI-nu/nu mice). The CIEA-NOG mice will, as they lack both NK-, T- and B-cells, have a lower amount of antibodies that can react against the transplanted cells, resulting in a slower capture of the transplanted cells in the spleen. It is, however, possible to use all 3 mouse strains in these homing experiments since the time span examined are relatively short as to avoid an immune response from the immune competent mice towards the xenotransplanted EOCs.

## Conclusion

In the present study, the effect of VDA treatment on EOC recruitment to the tumour in 3 different mice strains was examined. The recruitment and presence of EOCs at the tumour site of both untreated and VDA treated animals in the 3 different mice strains were demonstrated using ^111^In labelled EOCs. However the results did not demonstrate an increased recruitment of the xenotransplanted EOCs to tumour tissue 24 hours after treatment with CA4P or DMXAA.

## References

[1] Asahara T, Murohara T, Sullivan A, Silver M, van der Zee R, Li T, Witzenbichler B, Schatteman G, Isner JM (1997). Isolation of putative progenitor endothelial cells for angiogenesis. Science.

[2] Tura O, Skinner EM, Barclay GR, Samuel K, Gallagher RC, Brittan M, Hadoke PW, Newby DE, Turner ML, Mills NL (2013). Late outgrowth endothelial cells resemble mature endothelial cells and are not derived from bone marrow. Stem Cells.

[3] Nolan DJ, Ciarrocchi A, Mellick AS, Jaggi JS, Bambino K, Gupta S, Heikamp E, McDevitt MR, Scheinberg DA, Benezra R, Mittal V (2007). Bone marrow-derived endothelial progenitor cells are a major determinant of nascent tumor neovascularization. Genes Dev.

[4] Khakoo AY, Finkel T (2005). Endothelial progenitor cells. Annu Rev Med.

[5] Ingram DA, Caplice NM, Yoder MC (2005). Unresolved questions, changing definitions, and novel paradigms for defining endothelial progenitor cells. Blood.

[6] Timmermans F, Plum J, Yoder MC, Ingram DA, Vandekerckhove B, Case J (2009). Endothelial progenitor cells: identity defined?. J Cell Mol Med.

[7] Urbich C, Dimmeler S (2004). Endothelial progenitor cells: characterization and role in vascular biology. Circ Res.

[8] Shaked Y, Ciarrocchi A, Franco M, Lee CR, Man S, Cheung AM, Hicklin DJ, Chaplin D, Foster FS, Benezra R, Kerbel RS (2006). Therapy-induced acute recruitment of circulating endothelial progenitor cells to tumors. Science.

[9] Taylor M, Billiot F, Marty V, Rouffiac V, Cohen P, Tournay E, Opolon P, Louache F, Vassal G, Laplace-Builhe C, Vielh P, Soria JC, Farace F (2012). Reversing resistance to vascular-disrupting agents by blocking late mobilization of circulating endothelial progenitor cells. Cancer Discov.

[10] Ingram DA, Mead LE, Tanaka H, Meade V, Fenoglio A, Mortell K, Pollok K, Ferkowicz MJ, Gilley D, Yoder MC (2004). Identification of a novel hierarchy of endothelial progenitor cells using human peripheral and umbilical cord blood. Blood.

[11] Barclay GR, Tura O, Samuel K, Hadoke PW, Mills NL, Newby DE, Turner ML (2012). Systematic assessment in an animal model of the angiogenic potential of different human cell sources for therapeutic revascularization. Stem Cell Res Ther.

[12] Yoder MC, Mead LE, Prater D, Krier TR, Mroueh KN, Li F, Krasich R, Temm CJ, Prchal JT, Ingram DA (2007). Redefining endothelial progenitor cells via clonal analysis and hematopoietic stem/progenitor cell principals. Blood.

[13] Pagan J, Przybyla B, Jamshidi-Parsian A, Gupta K, Griffin RJ (2013). Blood outgrowth endothelial cells increase tumor growth rates and modify tumor physiology: relevance for therapeutic targeting. Cancers (Basel).

[14] Aicher A, Brenner W, Zuhayra M, Badorff C, Massoudi S, Assmus B, Eckey T, Henze E, Zeiher AM, Dimmeler S (2003). Assessment of the tissue distribution of transplanted human endothelial progenitor cells by radioactive labeling. Circulation.

[15] Mitchell AJ, Sabondjian E, Sykes J, Deans L, Zhu W, Lu X, Feng Q, Prato FS, Wisenberg G (2010). Comparison of initial cell retention and clearance kinetics after subendocardial or subepicardial injections of endothelial progenitor cells in a canine myocardial infarction model. J Nucl Med.

[16] Vaupel P, Kallinowski F, Okunieff P (1989). Blood flow, oxygen and nutrient supply, and metabolic microenvironment of human tumors: a review. Cancer Res.

[17] Carmeliet P, Jain RK (2000). Angiogenesis in cancer and other diseases. Nature.

[18] Kerbel R, Folkman J (2002). Clinical translation of angiogenesis inhibitors. Nat Rev Cancer.

[19] Siemann DW, Bibby MC, Dark GG, Dicker AP, Eskens FA, Horsman MR, Marme D, Lorusso PM (2005). Differentiation and definition of vascular-targeted therapies. Clin Cancer Res.

[20] Horsman MR, Siemann DW (2006). Pathophysiologic effects of vascular-targeting agents and the implications for combination with conventional therapies. Cancer Res.

[21] Dark GG, Hill SA, Prise VE, Tozer GM, Pettit GR, Chaplin DJ (1997). Combretastatin A-4, an agent that displays potent and selective toxicity toward tumor vasculature. Cancer Res.

[22] Murata R, Siemann DW, Overgaard J, Horsman MR (2001). Interaction between combretastatin A-4 disodium phosphate and radiation in murine tumors. Radiother Oncol.

[23] Tozer GM, Prise VE, Wilson J, Locke RJ, Vojnovic B, Stratford MR, Dennis MF, Chaplin DJ (1999). Combretastatin A-4 phosphate as a tumor vascular-targeting agent: early effects in tumors and normal tissues. Cancer Res.

[24] Murata R, Overgaard J, Horsman MR (2001). Comparative effects of combretastatin A-4 disodium phosphate and 5,6-dimethylxanthenone-4-acetic acid on blood perfusion in a murine tumour and normal tissues. Int J Radiat Biol.

[25] Tozer GM, Kanthou C, Baguley BC (2005). Disrupting tumour blood vessels. Nat Rev Cancer.

[26] Pettit GR, Singh SB, Hamel E, Lin CM, Alberts DS, Garcia-Kendall D (1989). Isolation and structure of the strong cell growth and tubulin inhibitor combretastatin A-4. Experientia.

[27] Baguley BC (2003). Antivascular therapy of cancer: DMXAA. Lancet Oncol.

[28] Roberts ZJ, Ching LM, Vogel SN (2008). IFN-beta-dependent inhibition of tumor growth by the vascular disrupting agent 5,6-dimethylxanthenone-4-acetic acid (DMXAA). J Interferon Cytokine Res.

[29] Greenberg DA, Jin K (2005). From angiogenesis to neuropathology. Nature.

[30] Hattori K, Dias S, Heissig B, Hackett NR, Lyden D, Tateno M, Hicklin DJ, Zhu Z, Witte L, Crystal RG, Moore MA, Rafii S (2001). Vascular endothelial growth factor and angiopoietin-1 stimulate postnatal hematopoiesis by recruitment of vasculogenic and hematopoietic stem cells. J Exp Med.

[31] Rafii S, Heissig B, Hattori K (2002). Efficient mobilization and recruitment of marrow-derived endothelial and hematopoietic stem cells by adenoviral vectors expressing angiogenic factors. Gene Ther.

[32] Iwaguro H, Yamaguchi J, Kalka C, Murasawa S, Masuda H, Hayashi S, Silver M, Li T, Isner JM, Asahara T (2002). Endothelial progenitor cell vascular endothelial growth factor gene transfer for vascular regeneration. Circulation.

[33] Overgaard J (1980). Simultaneous and sequential hyperthermia and radiation treatment of an experimental tumor and its surrounding normal tissue in vivo. Int J Radiat Oncol Biol Phys.

[34] Timmermans F, Van HF, De SM, Raedt R, Plasschaert F, De Buyzere ML, Gillebert TC, Plum J, Vandekerckhove B (2007). Endothelial outgrowth cells are not derived from CD133+ cells or CD45+ hematopoietic precursors. Arterioscler Thromb Vasc Biol.

[35] Bertelsen LB, Hagensen M, Busk M, Zhang R, Knudsen AS, Nielsen N, Falborg L, Moller BK, Horsman MR, Stodkilde-Jorgensen H (2014). In vivo bio-distribution and homing of endothelial outgrowth cells in a tumour model. Nucl Med Biol.

[36] Murata R, Overgaard J, Horsman MR (2001). Potentiation of the anti-tumour effect of hyperthermia by combining with the vascular targeting agent 5,6-dimethylxanthenone-4-acetic acid. Int J Hyperthermia.

[37] Iwami Y, Masuda H, Asahara T (2004). Endothelial progenitor cells: past, state of the art, and future. J Cell Mol Med.

[38] Bertolini F, Shaked Y, Mancuso P, Kerbel RS (2006). The multifaceted circulating endothelial cell in cancer: towards marker and target identification. Nat Rev Cancer.

[39] Horsman MR, Ehrnrooth E, Ladekarl M, Overgaard J (1998). The effect of combretastatin A-4 disodium phosphate in a C3H mouse mammary carcinoma and a variety of murine spontaneous tumors. Int J Radiat Oncol Biol Phys.

[40] Tozer GM, Kanthou C, Parkins CS, Hill SA (2002). The biology of the combretastatins as tumour vascular targeting agents. Int J Exp Pathol.

[41] Nielsen T, Murata R, Maxwell RJ, Stodkilde-Jorgensen H, Ostergaard L, Horsman MR (2008). Preclinical studies to predict efficacy of vascular changes induced by combretastatin a-4 disodium phosphate in patients. Int J Radiat Oncol Biol Phys.

[42] Lingen MW (2001). Role of leukocytes and endothelial cells in the development of angiogenesis in inflammation and wound healing. Arch Pathol Lab Med.

[43] Dudek AZ, Bodempudi V, Welsh BW, Jasinski P, Griffin RJ, Milbauer L, Hebbel RP (2007). Systemic inhibition of tumour angiogenesis by endothelial cell-based gene therapy. Br J Cancer.

[CR44] The pre-publication history for this paper can be accessed here:http://www.biomedcentral.com/1471-2407/14/903/prepub

